# Usher’s syndrome

**DOI:** 10.1016/S1808-8694(15)30113-0

**Published:** 2015-10-19

**Authors:** Maria Carolina Braga Norte, Antônio José Cortez Juares, José Carlos Nardi, Alfredo Rafael Dell’Aringa, Kazue Kobari

**Affiliations:** a Medical graduate, otorhinolaryngology resident, Marilia Medical School.; b Medical graduate, otorhinolaryngology resident, Marilia Medical School.; c Master’s degree, assistant professor of otorhinolaryngology, Marilia Medical School.; d Doctoral degree, head of the discipline of otorhinolaryngology, Marilia Medical School.; e Graduation, assistant professor of otorhinolaryngology, Marilia Medical School. Marilia Medical School.

**Keywords:** blindness, hearing loss, usher’s syndrome

## INTRODUCTION

Usher’s syndrome is an autossomal recessive disease^2^ characterized by pigmentary retinopathy (PR) and bilateral sensorineural hypoacusis.^4^ Von Graefe and Liebreich^3^ were the first to publish a paper describing the association between PR and deafness. The incidence is 3 to 4.4 per 100,000 people. The prevalence is 3% to 6% among auditory impaired people.4 It may be subdivided into four types of which the type II is the mildest form. Patients present with slowly progressive moderate bilateral sensorineural dysacusis that predominantly affects high frequencies; there is preservation of vestibular function. PR begins in adolescents or in young adults.1 The aim of this study was to describe and analyze the clinical presentation of Usher’s syndrome and to compare these findings with the current literature. We described a clinical case of a 33-year-old patient that presented with the type II of Usher’s syndrome. The patient signed a free informed consent form before participating in the study, according to the Resolution 196/96 - CNS/MS.

## CASE PRESENTATION

**Figure 1:**
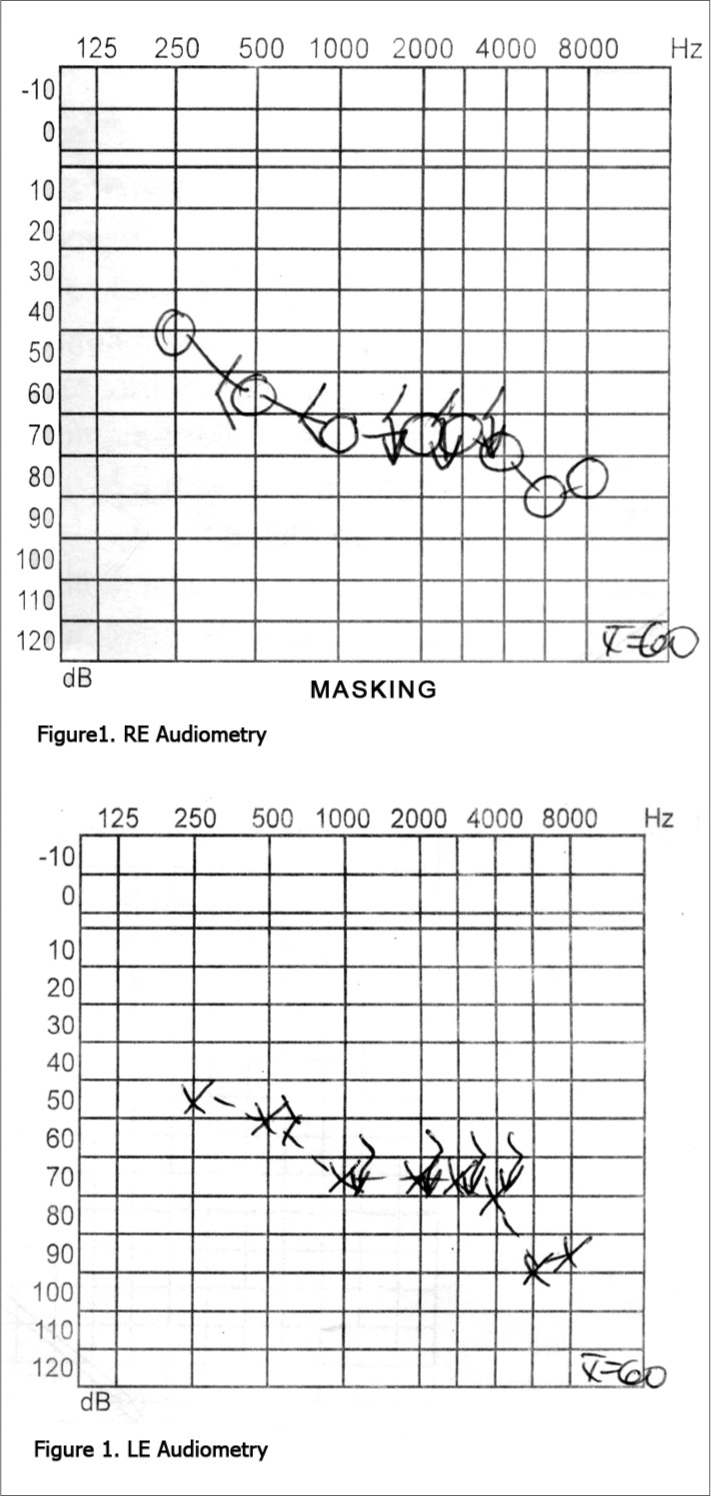
RE Audiometry

M. J. I., is a female, 33-year-old, white, married housewife, who concluded primary education. This patient presented with bilateral hypoacusis, which was worse in the left ear, since childhood; she also presented with impaired vision since she was age 16 years. Her parents were not close relatives. She has a 28-year-old sister, who also has bilateral hypoacusis since childhood that is more severe than that of the patient. The clinical ororhinolaryngological exam was within normal limits. The ophthalmologic test revealed right eye visual acuity only for hand movements, and left eye visual acuity only for light perception. Retinography showed bilateral hypopigmentation of the pigmentary epithelium, which included the macula. Fluorescent retinography showed sings of diffuse atrophy of the pigmentary epithelium, including the macula. The computerized visual field perimetry revealed a nearly complete absence of response to the stimulus in the right eye; this test was not done on the left eye due to low visual acuity. Pure tone audiometry showed bilateral moderate to severe sloping sensorineural hearing loss (Figure 1). The otoneurological exam suggested cervical peripheral labyrinth disease.

## DISCUSSION

Based on these data we concluded that the patient was a case of type II Usher’s syndrome, according to Merin et al.’s classification. Genetic counseling and follow-up was provided. A bilateral individual sound amplification device was recommended. An attempt was made to correct the visual deficit, with little success. Advances in genetic therapy are needed for improved therapeutic results.

## FINAL COMMENTS

We emphasize to ophthalmologists that any patient diagnosed with pigmentary retinopathy should be referred to the otorhinolaryngologist for an evaluation of auditory acuity, given that 10% of these patients will present with hearing loss. We also highlight the importance of an early diagnosis of Usher’s syndrome in order to attain better therapeutic results.
